# Effect of Fangxia-Dihuang Decoction on doxorubicin-induced cognitive impairment in breast cancer animal model

**DOI:** 10.3389/fonc.2025.1515498

**Published:** 2025-04-28

**Authors:** Xuan Wang, Qiqi Sun, Jianrong Li, Baoyong Lai, Xiaohua Pei, Nana Chen

**Affiliations:** ^1^ Beijing Obstetrics and Gynecology Hospital, Capital Medical University. Beijing Maternal and Child Health Care Hospital, Beijing, China; ^2^ The Third Affiliated Hospital of Beijing University of Chinese Medicine, Beijing, China; ^3^ The Xiamen Hospital of Beijing University of Chinese Medicine, Xiamen, China

**Keywords:** chemotherapy related cognitive impairment, breast cancer, Fangxia-Dihuang decoction, doxorubicin, traditional Chinese medicine

## Abstract

**Objective:**

Based on the murine model, this study explored the efficacy of Fangxia-Dihuang Decoction (FXDH) in interfering with cognitive impairment induced by doxorubicin (DOX) after chemotherapy for breast cancer.

**Methods:**

Build 4T1 breast cancer xenograft tumor model in Balb/c mice, intraperitoneal injection of DOX (5mg/kg) once a week, build the model of DOX induced chemotherapy related cognitive impairment (CRCI), and the administration lasted for three weeks. From the first week, while DOX was given, FXDH was given high, medium and low doses by gavage every day. Conduct Y-maze and Novel object recognition (NOR) tests, detect inflammatory factors and oxidative stress-related indicators in serum and hippocampus, observe neuroinflammation and neurodegenerative changes through immunofluorescence and Nissl staining. Observation of heart and liver injury through blood routine and cardiac Hematoxylin-Eosin(HE)Staining.

**Results:**

Administration of FXDH significantly improved cognitive impairment in mice. FXDH reduced the levels of pro-inflammatory cytokines IL-6, IL-12p70, and TNF-α (P<0.05), and increased the levels of anti-inflammatory cytokines IL-10 and IL-4 (P<0.05). FXDH increased the levels of GSH, GSH-PX, SOD, and CAT in serum and hippocampus (P<0.05), and decreased the level of MDA (P<0.05). The results of Nissl staining and immunofluorescence staining showed that FXDH improved the neurodegenerative lesions caused by DOX and the neuroinflammatory response in the hippocampus (P<0.05). The intermediate dose group of FXDH showed better efficacy. The results of blood routine and cardiac HE staining showed that compared with the 4T1 group, the serum ALT, AST, CK, LDH, and CKMB in DOX group mice were significantly increased (P<0.05). After FXDH administration, all indicators in mice were decreased, but there was no statistical difference. FXDH improved the disordered arrangement of myocardial cells, uneven cytoplasmic staining, and loose and disordered arrangement of myocardial fibers caused by DOX.

**Conclusion:**

In the animal model, FXDH has the effect of anti-cognitive impairment after chemotherapy for breast cancer, and can improve the DOX induced learning, memory and cognitive impairment in mice. FXDH can reverse DOX induced neuroinflammation by improving the neurodegenerative changes caused by DOX, reducing pro-inflammatory cytokine levels in mouse serum and hippocampus, increasing anti-inflammatory cytokine levels, and reducing oxidative stress response.

## Introduction

Cancer patients are prone to cognitive impairment after chemotherapy, mainly manifested as damage to their attention, learning ability, memory, etc., and can easily lead to accompanying symptoms such as emotional depression, anxiety, fatigue, sleep disorders, etc. (1), which can be temporary or permanent symptoms, and can remain stable or gradually worsen over time (2). At present, there are few epidemiological investigations on cognitive impairment after chemotherapy. According to literature reports, about 14% -85% of patients experience this side effect (3), which affects their quality of life (4). Many patients develop a fear of treatment after experiencing such symptoms and even demand termination of treatment (5).Weiss et al. published the first paper in 1974 revealing the cognitive impairment and neurotoxicity induced by chemotherapy drugs, and proposed the correlation between neurological problems in cancer patients and chemotherapy (6). In 1983, Silberfarb first proposed the concept of Chemotherapy related cognitive impairment (CRCI) (7), also known as the “chemotherapy brain” or “chemotherapy fog”, which vividly describes the cognitive and memory impairment that occurs in patients after chemotherapy. CRCI may exist in various cancers. Most studies focus on breast cancer, but it is also involved in other tumors. According to reports, CRCI can occur in colorectal cancer (8, 9), lung cancer (10, 11), testicular cancer (12–14), prostate cancer (15–18), ovarian cancer and other gynecological malignancies (18–20), hematological malignancies, especially after hematopoietic stem cell transplantation (21, 22).

At present, anthracyclines and taxanes are commonly used for chemotherapy of breast cancer, and now research has found that such chemotherapy drugs are more likely to lead to CRCI, so compared with other tumor patients, breast cancer patients are more likely to have CRCI. Neuroinflammation and oxidative stress are the main links in chemotherapy induced CRCI, and are associated with the progression of many neurodegenerative diseases, such as Parkinson’s disease, Alzheimer’s disease, etc. (4, 23). Due to the high demand for oxygen in the brain, it is highly susceptible to oxidative stress (24). According to reports, more than half of the chemotherapy drugs approved by the FDA induce oxidative stress in non-targeted tissues (25). This study selected DOX as the inducer of CRCI. DOX is a water-soluble molecule with a molecular weight of 580 daltons (26). It is widely believed that DOX has poor ability to penetrate the blood-brain barrier (BBB), but the drug can induce neurodegeneration by inducing systemic release of inflammatory mediators, which can penetrate the BBB and enhance neuroinflammation and oxidative stress response (27).

Modern research has shown that DOX has direct and indirect neurotoxicity. DOX induces neurotoxicity by enhancing the production of ROS and causing depolarization of the mitochondrial membrane in neurons (28). Research demonstrated that DOX accumulates within neuronal nuclei, resulting in DNA double-strand breaks (DSBs) and cross-linking of DNA (29). At the same time, DOX may disrupt the neuronal degradation pathway of progenitor cells, impair lysosomal function, promote the formation of pre autophagic structures, enhance autophagy, and affect the clearance of autophagy marker protein p62 (30). In addition, studies have found that animals treated with DOX exhibit significantly reduced neurogenesis (31), affect neurotransmitter levels (32–34), and lead to abnormal neural signal transduction (35), resulting in cognitive impairment. It has been documented that DOX can induce epigenetic reprogramming, which is another crucial mechanism potentially underlying persistent cognitive decline (36). Studies indicated that DOX, a quinone-based compound, possesses a structure susceptible to single-electron reduction. It can be converted into semiquinone radicals by enzymes such as NADPH cytochrome P450 reductase (37), NADH dehydrogenase (mitochondrial complex I) (37), and cytoplasmic xanthine oxidase (38). The semiquinone form of doxorubicin reacts with oxygen, reverting to its natural quinone state while generating a superoxide anion radical (O2•−) (39). The superoxide anion radical is a secondary ROS species, which can further produce hydrogen peroxide and hydroxyl radicals, leading to peripheral oxidative stress and inducing CRCI (39).

For CRCI intervention, there are mainly two treatment methods: medication and non-medication. Non pharmacological therapies mainly include Cognitive Behavioral Therapy (CBT) (40–43), physical and mental intervention (40, 44–47), exercise (48–51), human care (52, 53), etc. At present, there is no unified drug treatment method for CRCI. Most treatment methods are still in the exploratory stage of CRCI intervention. FXDH represents a modified formulation derived from the Fangji-Dihuang decoction, a traditional prescription documented in the Golden Chamber, a seminal text in Traditional Chinese Medicine (TCM). While the original formulation has historically been employed for managing emotional disturbances, especially those associated with liver and kidney Yin-deficiency, phlegm accumulation, and blood stasis based on TCM principles (54, 55), the enhanced decoction incorporates additional medicinal herbs including Prunella vulgaris, Gardenia jasminoides, and Pinellia ternata to augment its therapeutic efficacy. In clinic, we often use it as adjuvant therapy for breast cancer. We found that FXDH has a good therapeutic effect on breast cancer patients with depression, anxiety, insomnia, and cognitive decline, and could alleviate nausea and vomiting and other side effects caused by chemotherapy. The composition of FXDH is: Radix Rehmanniae (Sheng Di), Stephania tetrandra (Fang Ji), Selfheal (Xia Ku Cao), Cassia twig (Gui Zhi), Licorice (Gan Cao), Radix Sileris (Fang Feng), Fructus Gardeniae (Zhi Zi), Pinellia ternata (Ban Xia), Rhizoma Zingiberis (Gan Jiang). In the preliminary research, the team applied high-performance liquid chromatography (UPLC-HRMS) to analyze the components of FXDH, and the results showed that the decoction had standard production processes and data (56). Consequently, a total of 32 bioactive constituents were identified in FXDH, predominantly comprising aporphine alkaloids, benzopyran derivatives, morphinan compounds, protoberberine-type alkaloids, flavonoid glycosides, phenolic acids, cinnamic acid derivatives, glycerophospholipids, saccharolipids, carboxylic acids, and fatty acyl compounds (56). Animal and cell experiments showed that FXDH could improve the depression of mice and had the effect of anti-breast cancer (56).

Pharmacological research has found that there are many components in the herb composition of FXDH that have protective effects on the brain and cognitive function. The study found that tetrandrine (Tet), the extract of Stephania tetrandra, the main drug of FXDH, has the effect of antagonizing streptozocin induced hippocampal damage in diabetes mice, and its mechanism may be related to inhibiting its oxidative stress and Smad signaling pathway (57). In a rat model, Tet can significantly improve learning and memory impairment caused by vascular dementia injury, and its mechanism may be related to downregulating the expression of S100B in astrocytes, thereby inhibiting brain damage caused by inflammatory response in the hippocampus of vascular dementia rats (58). Radix Rehmaniae can alleviate cognitive impairment and brain histopathological changes in Alzheimer’s disease mice. The mechanism may be related to the regulation of the INSR/IRS-1/AKT/GSK-3 β signaling pathway and gut microbiota (59). Another study suggests that Gui Zhi is a promising neuroprotective agent that can alleviate neurotoxicity caused by other herb (60). Licochalcone A is the main component of licorice, which shows the ability to reduce amyloid plaques, a hallmark of Alzheimer’s disease, and exhibits antioxidant properties by activating nuclear factor erythroid 2-related factor 2 (NRF2) (61). Based on this, this study intends to explore the role of FXDH in preventing cognitive impairment after chemotherapy for breast cancer based on animal experiments, so as to provide a basis for its clinical application and Traditional Chinese Medicine intervention in CRCI.

## 2 Materials and methods

### 2.1 Materials and reagents

Doxorubicin (DOX), purchased from manufacturer Apexbio (A3966), The malondialdehyde (MDA) assay kit (A003-1-2), total superoxide dismutase (T-SOD) assay kit (A001-1-2), catalase (CAT) assay kit (A007-1-1), glutathione peroxidase (GSH-PX) assay kit (A005-1-2), and total glutathione (T-GSH) assay kit (A061-2-1) were all purchased from Nanjing Jiancheng Bioengineering Institute (Nanjing, China). All raw medicinal materials of FXDH are provided by the Pharmacy Department of Xiamen Traditional Chinese Medicine Hospital (Xiamen, China).

### 2.2 Dosage selection of DOX

Dissolve DOX (0.5 mg/ml) in 0.9% phosphate buffered saline (PBS; 20 mmol/L sodium phosphate; 150 mmol/L sodium chloride; pH=7.4), adjust the pH to 6.8 with NaOH (62–65). In this study, we chose to administer DOX via intraperitoneal injection once a week at a dose of 5mg/kg for 3 times (The reasons for dose selection can be found in **Supplementary Material 6**).

### 2.3 Preparation of FXDH

The detailed composition of FXDH is: Radix Rehmanniae (Sheng Di) 30g, Stephania tetrandra (Fang Ji) 10g, Selfheal (Xia Ku Cao) 15g, Cassia twig (Gui Zhi) 6g, Licorice (Gan Cao) 6g, Radix Sileris (Fang Feng) 10g, Fructus Gardeniae (Zhi Zi) 10g, Pinellia ternata (Ban Xia) 9g, Rhizoma Zingiberis (Gan Jiang) 9g. Weigh 4 times the amount of medicine material crude slices, totaling 420 g (105 g * 4 = 420g), and extract them by reflux extraction at 90 °C for 3 times (1.5 hours each time) in a ratio of 1:12 of raw medicinal materials: distilled water. After that, combine the filtrate and evaporate it in a 60 °C oven to obtain the extract. Dry it under reduced pressure to obtain a dry powder, with approximately 2.44 g of raw medicinal materials per 1 g of dry powder. Quantitatively divide the prepared drug powder into centrifuge tubes, sterilize by irradiation, and store it in a cool, dry, and clean environment for future use. The FXDH group was administered daily by gavage using a solution prepared from FXDH freeze-dried powder. The dosage of each administration was as follows:

Each person takes one dose per day, which is 105 g/day. Based on the human equivalent dose calculation based on body surface area, it is calculated based on an adult weight of 70 kg, The weight of each mouse is calculated as 0.02 kg. Each medium dose group mouse was given a daily dose of raw medicinal materials = [9.1 * (105 g/70 kg)] * 0.02 kg=13.65 g/kg · d * 0.02kg=0.273 g. According to the above concentration ratio, it was equivalent to 0.112 g/day of dry powder. Each mouse is orally administered once a day, 0.2 ml each time, resulting in a drug concentration of 0.56 g/ml. Similarly, the low-dose group of mice was administered at a concentration of 0.28 g/ml. Due to experimental limitations (see **Supplementary Material 6** for details), the concentration of the high-dose group was set at 0.78 g/ml.

### 2.4 Cultivation of 4T1 cells

4T1 breast cancer cell line, purchased from Jiangsu Kaiji Biotechnology Co., Ltd., article number: KG338. The cells were cultured in RPMI 1640 medium (Gibco, Brazil) containing 10% inactivated fetal bovine serum and grown in an incubator with 5% CO2 and 37°C.

### 2.5 Experimental animals

6-8 week old female Balb/c mice, purchased from Beijing Weitong Lihua Experimental Animal Technology Co., Ltd. (Beijing, China), license number: SYXK (Beijing) 2020-0033, kept in the animal room of Beijing University of Traditional Chinese Medicine (BUCM), license number: SYXK (Beijing) 2023-0011.All experimental procedures were strictly conducted in accordance with the “Guidelines for the Management and Use of Experimental Animals” issued by the Ethics Committee for the Welfare of Experimental Animals at Beijing University of Traditional Chinese Medicine. This study has passed the animal ethics review of BUCM, with ethics number: BUCM-2023032016-1158 23.4.12.

### 2.6 Experimental design

**Figure 1:**
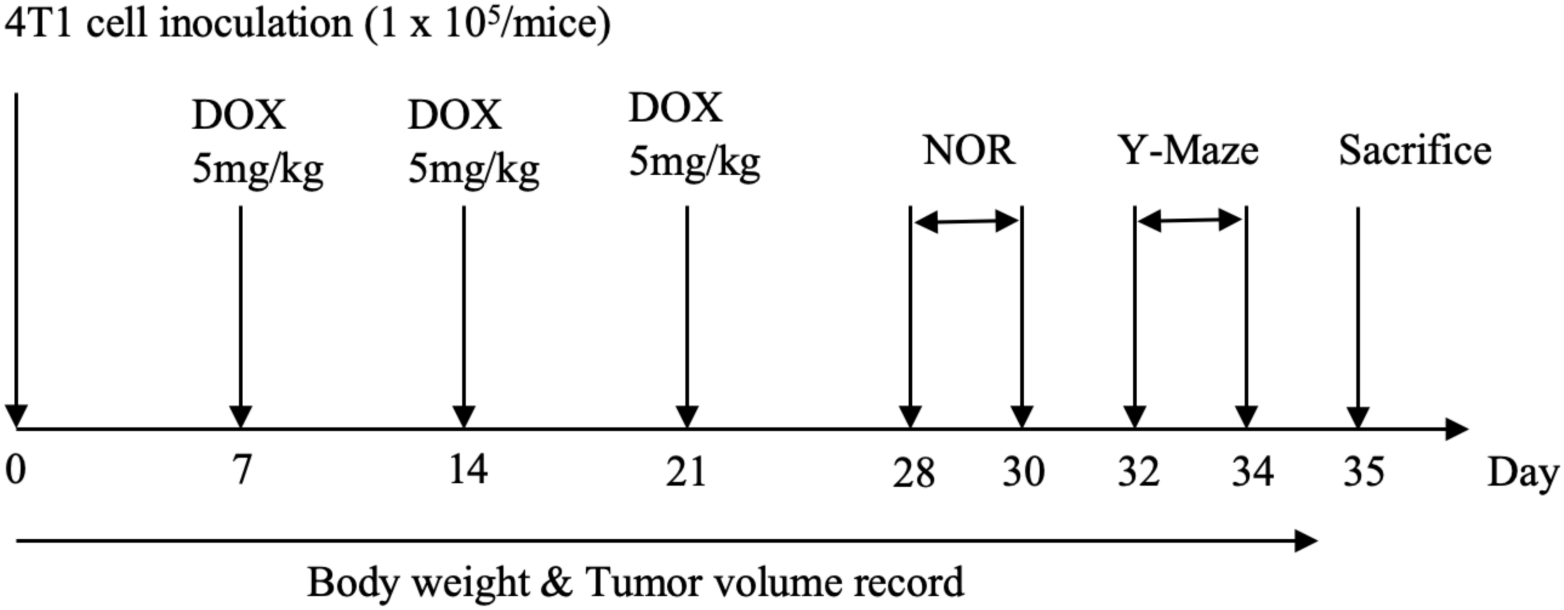
Experimental flowchart.

After one week of adaptive feeding, mice were randomly divided into six groups: blank group (Blank), tumor-bearing group (4T1), DOX tumor-bearing group (DOX), DOX+FXDH high-dose tumor-bearing group (DOX+FXDH1), DOX+FXDH medium dose tumor-bearing group (DOX+FXDH2), and DOX+FXDH low-dose tumor-bearing group (DOX+FXDH3). In addition to the blank group, the mice in the other groups were used to construct breast cancer transplantation tumor models with 4T1 cells. Animals in the DOX group and DOX+FXDH (1-3) groups received weekly intraperitoneal injections of 5 mg/kg DOX. The Blank group, 4T1 group, and DOX group were orally administered sterile distilled water once a day for 3 consecutive weeks. The drug intervention methods for each group of mice are shown in **Table 1**, and the experimental process in this section is shown in **Figure 1**.

**Table 1:** Drug intervention measures for each group.

Grouping	Handling method
Blank	Weekly intraperitoneal injection of physiological saline+daily gavage of distilled water
4T1	Weekly intraperitoneal injection of physiological saline+daily gavage of distilled water
DOX	DOX intraperitoneal injection once a week+daily distilled water gavage
DOX+FXDH1	DOX intraperitoneal injection once a week+high-dose FXDH daily gavage
DOX+FXDH2	DOX intraperitoneal injection once a week+moderate dose FXDH daily gavage
DOX+FXDH3	DOX intraperitoneal injection once a week+low-dose FXDH daily gavage

There are a total of 90 experimental mice in this section, with 15 mice in each group. In addition to Blank group, 75 mice were used to establish tumor bearing breast cancer models. Randomly divide all mice into 6 groups.

### 2.7 General observation and measurement of tumor growth indicators

This experiment dynamically observed the mental, activity, and gait status of mice in each group during the experiment period, and recorded the weight of mice every week. Using 3.0 * 3.0 mm as the criterion for tumor formation, it is determined that the modeling is successful. Every 3 days, tumor size was measured using a vernier caliper, and tumor volume was calculated using the following equation (56): Volume(mm^3^)=0.5 * [length(mm)] * [width (mm)] ^2^.After anesthetizing and euthanizing the mice, the subcutaneous tumor tissue of the mice was removed, and the tumor morphology of each group of transplanted tumors was photographed and recorded. The tumor mass of each group of mice was weighed, and the average tumor tissue mass of each group of mice was calculated. The tumor inhibition rate was calculated according to the following formula: Tumor inhibition rate= (average tumor weight of model group - average tumor weight of medication group)/(average tumor weight of model group) * 100%. During the experiment, a humane endpoint was established according to relevant ethical regulations in the laboratory. If the weight of tumor bearing mice decreased by more than 20%, or if the tumor diameter was greater than 1.5 cm or ulceration caused significant pain, euthanasia should be performed in a timely manner to alleviate animal suffering.

### 2.8 Behavioral experiment

#### 2.8.1 Y maze

The Y maze is mainly used to test the identification ability, learning ability, and memory of animals (66). The Y maze consists of three arms of identical size and space, and a central connecting area (arm length: 40 cm, arm bottom width: 3 cm, arm upper width: 13 cm, height of wall: 15 cm, BrainScience Idea, Osaka, Japan). The angle between adjacent arms is 120°. The specific experimental operation is as follows: Each mouse was placed in the end of one arm and record the order in which the mouse enters each arm within 10 minutes. Spontaneous rotation (Alternation) is defined as the continuous entry of mice into three arms, such as (1, 2, 3 or 1, 3, 2). Calculate spatial cognition as measured by spontaneous alternation: the number of successful alternations/(the total number of entries – 2) (67). After each experiment, the maze was cleaned with disinfectant solution. Their arm entries and alterations were monitored over a 10-minute period using the EthoVision XT video tracking system (68).

#### 2.8.2 Novel object recognition

NOR is one of the common behavioral experiments for evaluating cognitive function, commonly used to assess the memory and learning abilities of mice. The device for NOR is a wooden open box with a length * width * height of 30cm * 30cm * 40cm. Before testing, let the mice adapt to the environment in the experimental room for 1 hour, keep the room quiet and tidy, and adjust the indoor lighting (soft dark light). This experiment is divided into two stages: familiarization period and testing period. During the familiarization period experiment, two identical objects (A and B) were symmetrically placed in the box at a distance of 5 cm from the box wall. Before the experiment began, each mouse was placed with its back facing the object and allowed to explore freely for 10 minutes. The activity of the mice was recorded in a video, and the time they spent exploring the two objects (TA and TB) was counted separately. Climbing, sniffing, or licking objects with their forelimbs was defined as exploration objects, while lying still on the object could not be considered as exploration behavior. Then move the mice back into the cage and enter the testing phase after 1 hour: replace one of the two objects with a completely different (shape, material, etc.) object (C), and follow the same procedure as during the familiarization phase. Record the exploration behavior of animals towards new and old objects for 5 minutes. After each mouse is tested, it should be thoroughly cleaned by wiping the bottom, inner walls, and objects of the box with 70% alcohol. The preference index is used to evaluate the behavior of mice, and the calculation formula is: New item preference index=time spent exploring new objects/(time spent exploring new objects + time spent exploring old objects) * 100% (69).

### 2.9 Cytokine detection

24 hours after the behavioral test, the mice were deeply anesthetized with pentobarbital sodium (100 mg/kg, intraperitoneal injection, Sigma Chemical Company, MO, USA). When the mouse loses consciousness, blood is collected by cardiac puncture and stored in a 1.5 ml Eppendorf tube. The tube is centrifuged at 3500 r/min for 7 minutes to separate the serum. Immediately after cardiac perfusion with cold phosphate buffered saline (PBS), the brains of mice were removed. Dissect the right hippocampus from the brain. Serum and hippocampus specimens intended for cytokine analysis were stored at −80°C prior to processing. The concentrations of IL-6, IL-10, TNF-α, IL-12p70, IL-4 and IL-10 were determined utilizing the Cytometric Bead Array (CBA) method (CAT#740827, Biolegend, USA). The prepared samples and standards were quantified using the specialized software provided by CBA.

TBA method was used to detect MDA, hydroxylamine method was used to detect SOD, visible light method was used to detect CAT, colorimetric method was used to detect GSH-PX, and spectrophotometric method was used to detect total glutathione GSH. Follow the instructions in the reagent kit manual for the operation steps.

### 2.10 Nissl staining observation of neuronal morphology

Remove the left hemisphere of the mouse from the brain and fix it in 4% paraformaldehyde for 24 hours, then incubate it in 30% sucrose for 48 hours. Use Thermo NH50 low-temperature thermostat to take hippocampal coronal sections with a thickness of 16μm, and use 0.5% crystal violet for Nissl staining. Two independent observers observed the structural and pathological changes of neurons at different locations in the hippocampus under a 200x magnification optical microscope.

### 2.11 Immunofluorescence staining and analysis of GFAP and IBA-1

The remaining sections were used for immunofluorescence staining of glial fibrillary acidic protein (GFAP, reactive astrocytes), ionized calcium binding adapter molecule 1 (IBA-1, microglia), and 4’, 6-diamidino-2-phenylindole (DAPI, nucleus). After blocking with PBS containing 5% normal goat serum, 1% bovine serum albumin, and 0.5% TritonX, GFAP rabbit polyclonal antibody (1:500; Proteintech, 23935-1-AP) and IBA1 Rabbit polyclonal antibody (1:2000; Proteintech,10904-1-AP) were used overnight at 4°C. On the second day, the slices were incubated with a fluorescent dye conjugated secondary antibody (goat anti rabbit, 1:200; Servicebio) at room temperature for 1 hour, and continuously incubated with DAPI (0.01mol/L in PBS, Beyotime, C1005) at room temperature for 10 minutes, then washed in PBS and installed. The fluorescence image was captured on a NIKON Eclipse CI microscope. Each hippocampus was magnified 400 times, and 5-6 representative images were captured and analyzed using ImageJ by researchers.

### 2.12 Collection and preparation of cardiac tissue samples

Heart tissue was perfused with PBS, fixed with 4% paraformaldehyde for 24 hours, embedded in paraffin, and then sliced horizontally to a thickness of 5μm. HE staining is used to evaluate pathological damage of myocardial tissue. Measure images using an automatic image analysis system (Image Pro Plus 5.0).

### 2.13 Data statistical analysis

All statistical analyses were conducted using SPSS 26.0, and GraphPad Prism 8.0 was utilized to generate the graphs. Two-way or one-way analysis of variance was used to determine p-values, followed by a *post hoc* analysis with the least significant difference in multiple comparison tests. The statistical significance threshold was set at P <0.05.

## 3 Results

### 3.1 General state and weight

**Figure 2:**
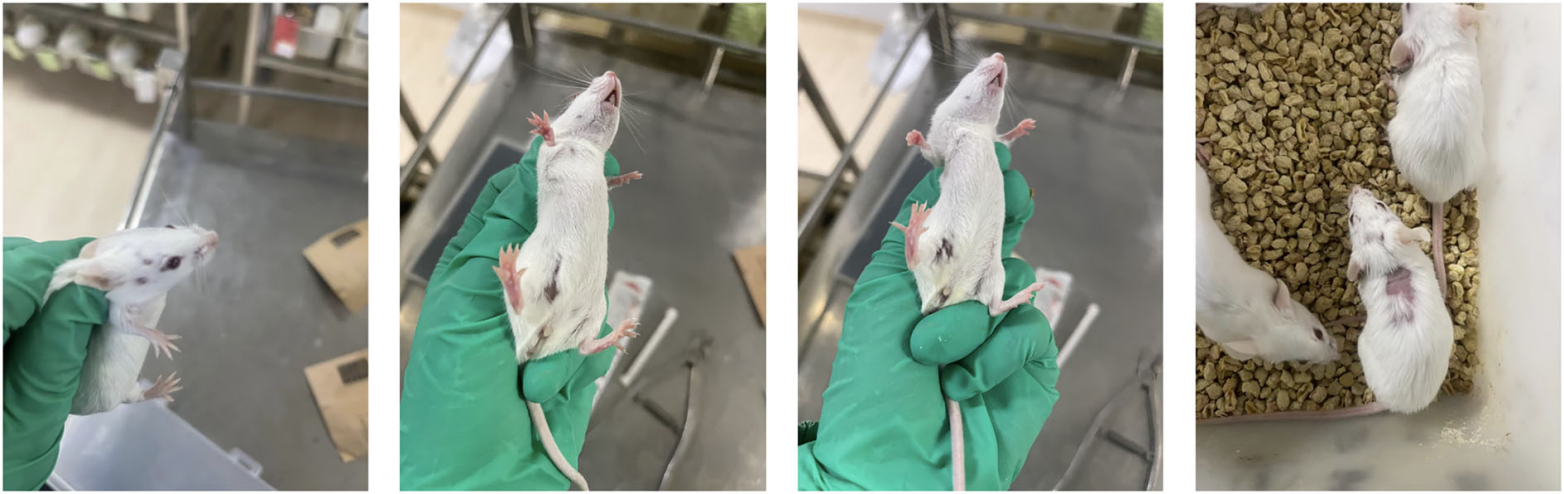
Hair loss phenomenon in mice after DOX administration. After the second intraperitoneal injection of DOX, most mice showed varying degrees of hair loss, with hair loss mostly occurring in the head, back, and abdomen (which may be related to the injection site of DOX).

This experiment first observed and recorded the general state and body weight of each group of mice. All groups of mice showed sensitive reactions, obvious grasping resistance, neat hair luster, normal stool morphology, moderate dryness and wetness, and yellow urine. Throughout the entire experimental period, no significant changes were observed in the general state of the blank group mice. On the 7th day of the experiment, except for the Blank group, a raised mass of approximately 3mm * 3mm appeared around the fourth pair of nursing pads on the left side of all other groups of mice, and no significant changes were observed in their general condition. Subsequently, the mice in each group gradually showed dull and dull hair, with significant differences in appearance compared to the blank control group mice; In addition, each group of mice also showed varying degrees of emaciation and arching of the back. Compared with the Blank and 4T1 groups, after the second intraperitoneal injection of DOX, both the DOX group and the DOX+FXDH (1-3) group showed varying degrees of abnormal behaviors such as increased sustained activity, non-stop running, abnormal excitement (see **Supplementary Material 1**), and aggression in mice, with the DOX and DOX+FXDH1 groups showing the most significant changes. It is worth noting that about 14 days after the start of the experiment, both the DOX group and DOX+FXDH (1-3) mice showed varying degrees of hair loss, mainly in the head, neck, back, and abdomen (**Figure 2**).

**Figure 3:**
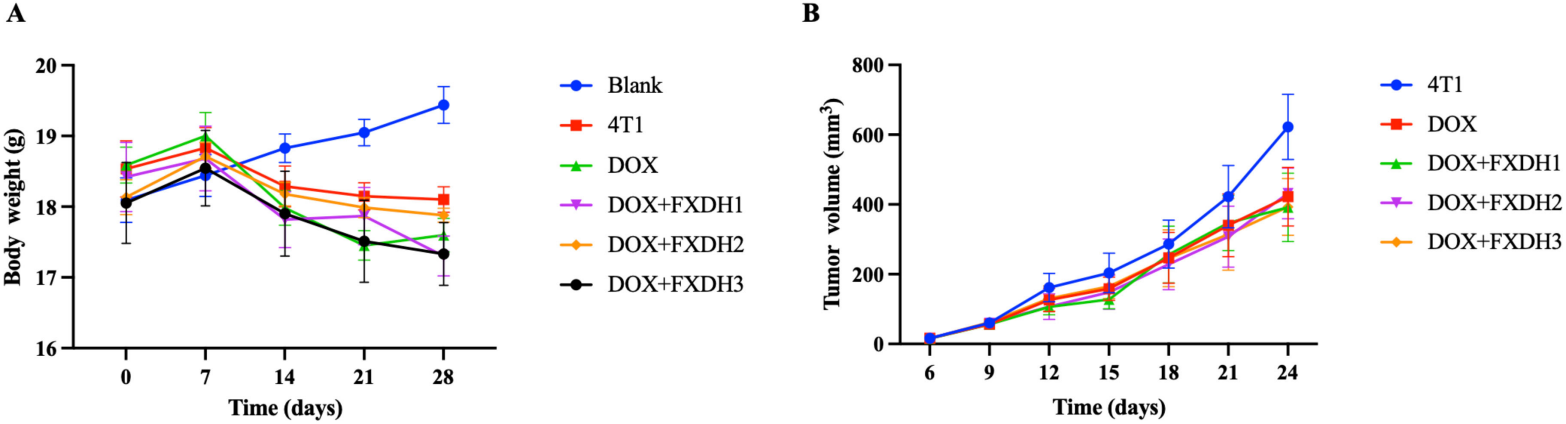
Changes in body weight and tumor volume. **(A)**. Measure the weight of mice every 7 days from day 0 to day 28 Weight change trend of mice in each group (n=6). **(B)**. After successful modeling, the longitudinal and transverse diameters of mouse tumors were measured every 3 days and the tumor volume was calculated. Tumor growth curve of tumor bearing mice (n=5).

We calculated the weight changes of mice at different time points (0 days, 7 days, 14 days, 21 days, and 28 days). Before modeling and drug intervention, the weight of mice in each group was basically balanced, and the difference was not statistically significant. Throughout the entire experiment, the weight of the Blank group mice showed a continuous upward trend, while the weight of the other groups of mice showed an overall downward trend from day 7 to day 28. Compared with the 4T1 group, the DOX+FXDH1 group and DOX+FXDH3 group showed significant weight loss at 28 days, with statistical differences (18.10 ± 0.44 vs 17.30 ± 0.70, and 18.10 ± 0.44 vs 17.33 ± 0.44, P<0.05). However, at the same time point, compared with the 4T1 group, there was no significant difference in weight between the DOX group and the DOX+FXDH2 group. Except for the Blank group, there was no statistically significant difference in weight changes between the other groups compared to the DOX group. The changes in body weight of each group of mice over time are detailed in **Supplementary Materials 2** and **Figure 3**.

### 3.2 Tumor growth in mice

#### 3.2.1 Changes in tumor volume

About 7 days after inoculation of 4T1 breast cancer cells, each tumor bearing mouse can reach a tumor of about 3 * 3mm in size, which is hard, indicating that the inoculation of breast cancer cells was successful, and the tumor size of each group of tumor bearing mice was basically balanced, with no statistically significant difference. From day 6 to day 27, there was a positive correlation between tumor volume size and time in all groups. Except for group 4T1, there was no statistical difference between the groups (P>0.05), and the trends were similar among the groups. On the 12th day, the tumor volume of the DOX+FXDH1 and DOX+FXDH2 groups was significantly lower than that of the 4T1 group (P<0.05), while there was no statistical difference between the DOX group, DOX+FXDH3 group, and 4T1 group. On days 24 to 27, the tumor volume of mice in the DOX group and DOX+FXDH (1-3) group was significantly lower than that in the 4T1 group (P<0.05). The changes in tumor volume over time in tumor bearing mice are shown in **Supplementary Materials 3** and **Figure 3**.

#### 3.2.2 Tumor weight and inhibition rate

On the 35th day of the experiment, mice were anesthetized and euthanized. The tumor weight of each group of tumor bearing mice was measured and the tumor inhibition rate was calculated. The results are shown in **Supplementary Material 4**. Compared with the 4T1 group mice bearing tumors alone, the tumor weight of the DOX+FXDH1 group and DOX+FXDH2 group mice was significantly reduced (1.26 ± 0.24 g vs 0.93 ± 0.27g, 1.26 ± 0.24 g vs 0.80 ± 0.13, P<0.05), with tumor inhibition rates of 26.19% and 36.51%, respectively. The DOX+FXDH2 group had the highest tumor inhibition rate, while the other groups showed certain tumor inhibition effects, but there was no statistical difference in tumor weight compared to the 4T1 group mice.

### 3.3 Y-maze testing of learning and memory abilities in mice

**Figure 4:**
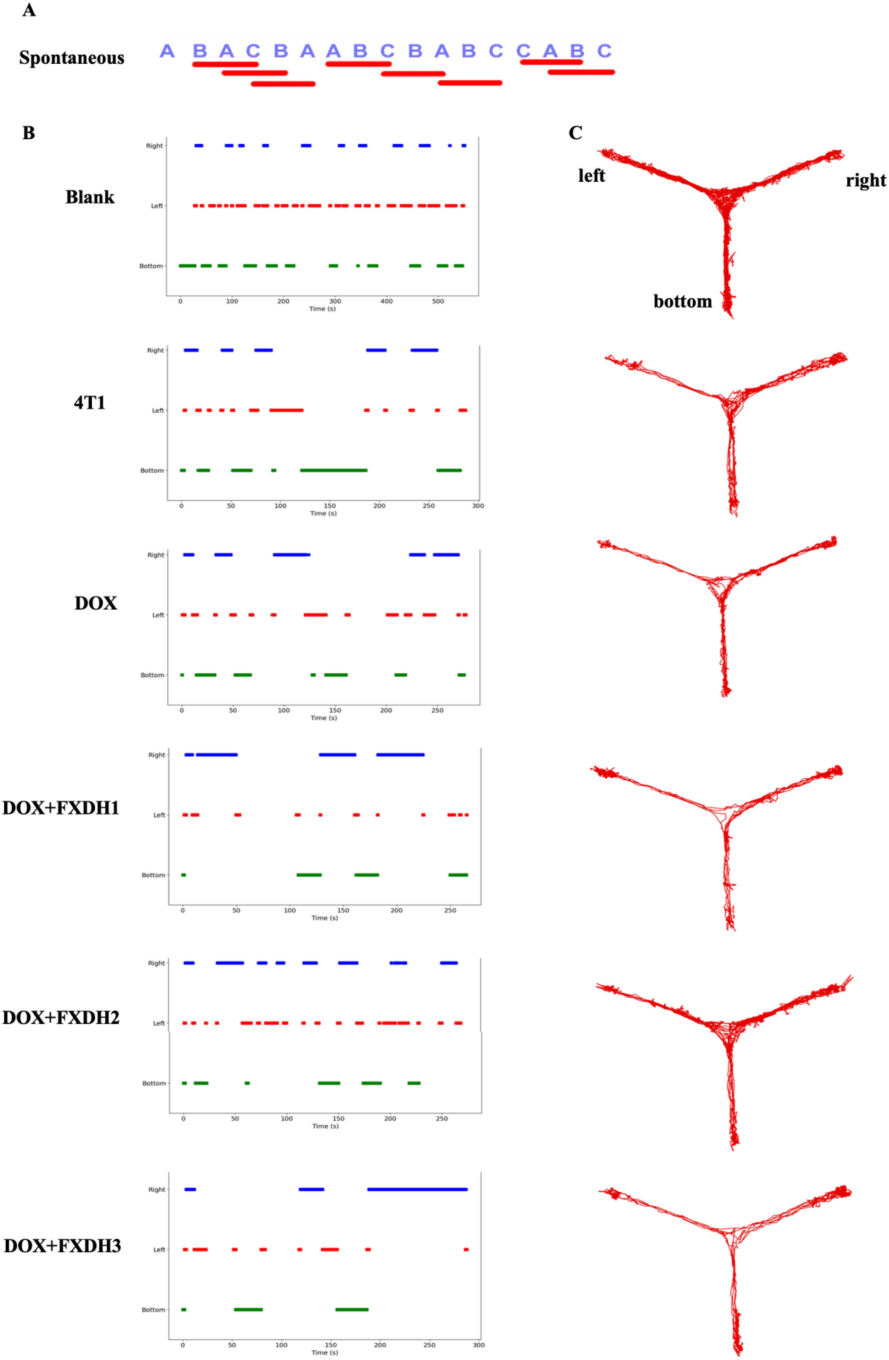
Movement trajectory and percentage of spontaneous alternations changes of Y-maze mice (n=6). **(A)** Schematic diagram of spontaneous alternations in Y maze; **(B)** The time distribution of mice entering the Y maze with three arms; **(C)** The movement trajectory of mice in the Y maze.

**Figure 5:**
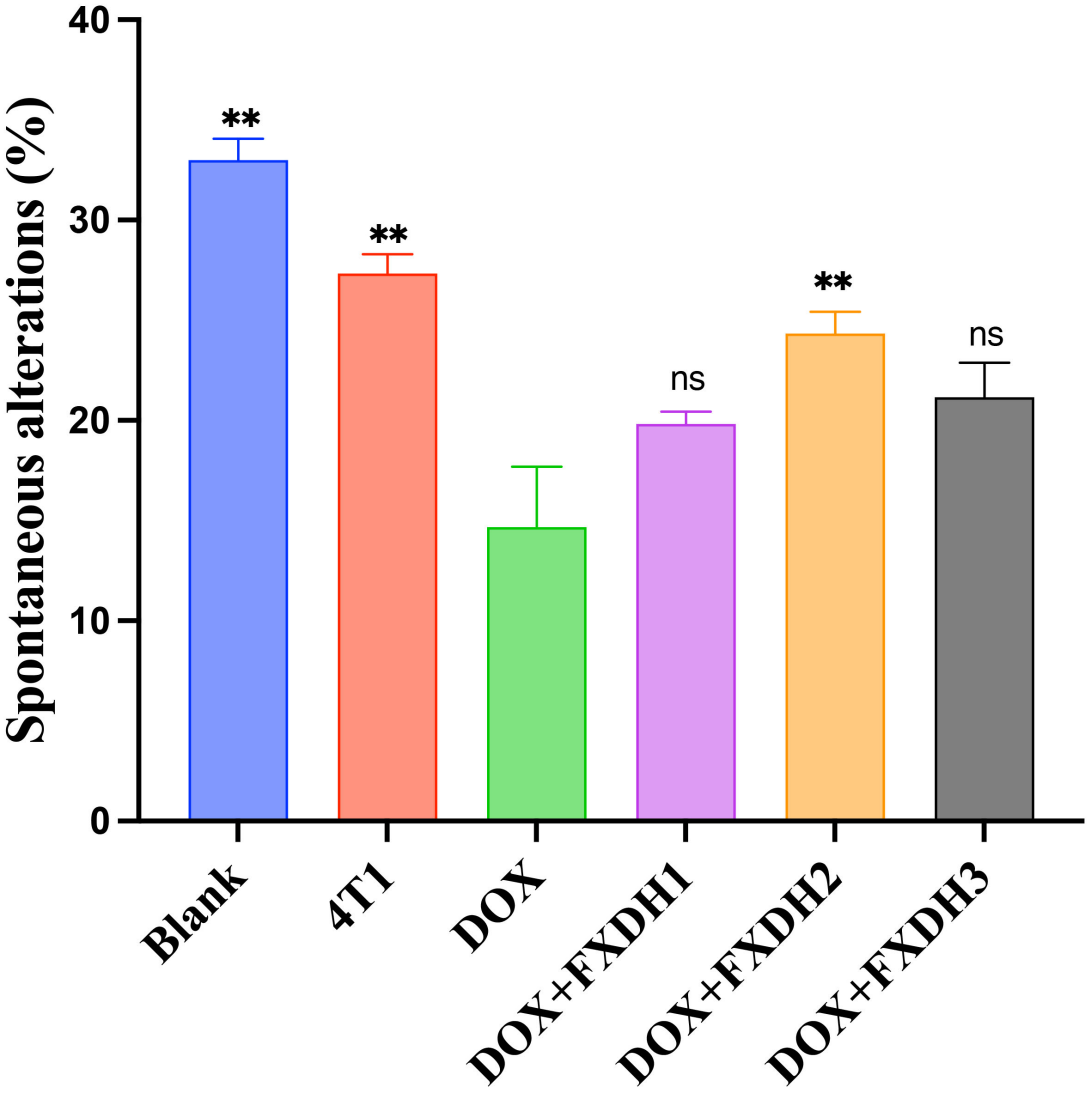
Statistical chart of the percentage of spontaneous alterations in Y-maze (n=6). ** Indicates that compared with the DOX group, ** P<0.01, ns represents no statistically significant difference.

In this study, we used the Y-maze to evaluate the intervention of each group on the learning and memory abilities of mice. Analyze and statistically analyze the spontaneous alternations percentage of each group of mice in the Y maze experiment (n=6, 6 groups, a total of 36 mice). The results showed that compared with the Blank group, the DOX group had a significantly lower percentage of spontaneous alternations (P<0.01), and the 4T1 group had a lower percentage of spontaneous alternations than the Blank group, but no statistical difference was shown. This result indicates that in this experiment, the tumor itself can reduce the cognitive and learning abilities of mice, but the difference is not statistically significant. The percentage of spontaneous alternations in the DOX+FXDH2 group of mice was significantly increased compared to the DOX group (P<0.01), while the percentage of spontaneous alternations in the DOX+FXDH1 and DOX+FXDH3 groups of mice was slightly increased, but there was no significant statistical difference compared to the DOX group (**Figures 4**, **5**).

### 3.4 Evaluation of cognitive function in mice through NOR

**Figure 6:**
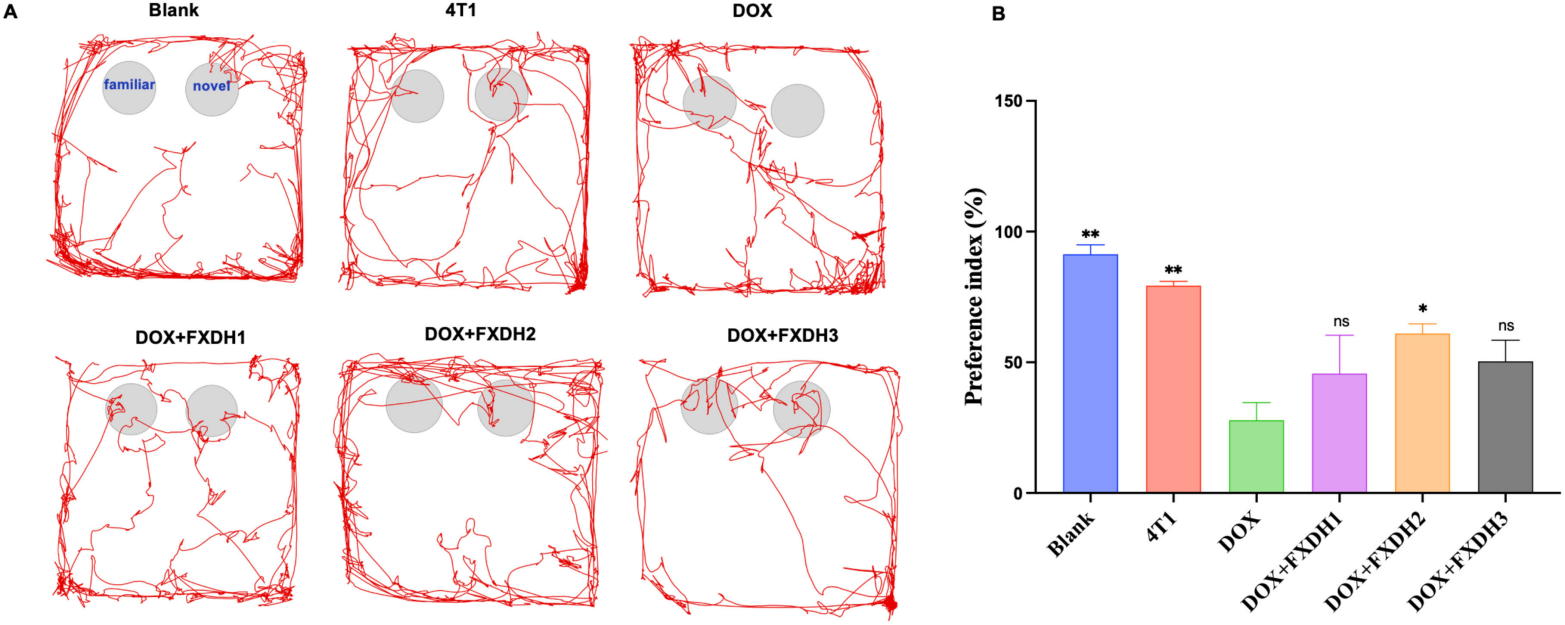
Movement trajectories and statistical chart of preference index (n=6). **(A)**. The NOR experiment is divided into a familiarization period and a testing period, as shown in the movement trajectory of the blank group. During the testing period, if mice show more exploratory behavior towards new objects, it indicates stronger memory and learning abilities. **(B)**. * Indicates that compared with the DOX group, * P<0.05, ** P<0.01, ns represents no statistically significant difference.

The movement trajectory of the mice in the new object recognition experiment is shown in **Figure 6A**. Compared with the Blank group and 4T1 group, the DOX group showed a significant decrease in the preference index (P<0.01). Compared with the Blank group, the 4T1 group had a slightly lower preference index, but no statistical difference was observed. This result indicates that under the experimental conditions, the tumor reduces the cognitive, memory, and learning abilities of mice, but the difference is not statistically significant. Compared with the DOX group, the DOX+FXDH2 group showed a significant increase in the preference index (P<0.05), while the DOX+FXDH1 and DOX+FXDH3 groups showed an increase in the preference index compared to the DOX group, but the difference was not statistically significant (**Figure 6B**). The results indicate that the FXDH medium dose group can improve the cognitive, learning, and memory deficits induced by DOX in mice.

### 3.5 CBA method for evaluating cytokine levels in serum and hippocampus

**Figure 7:**
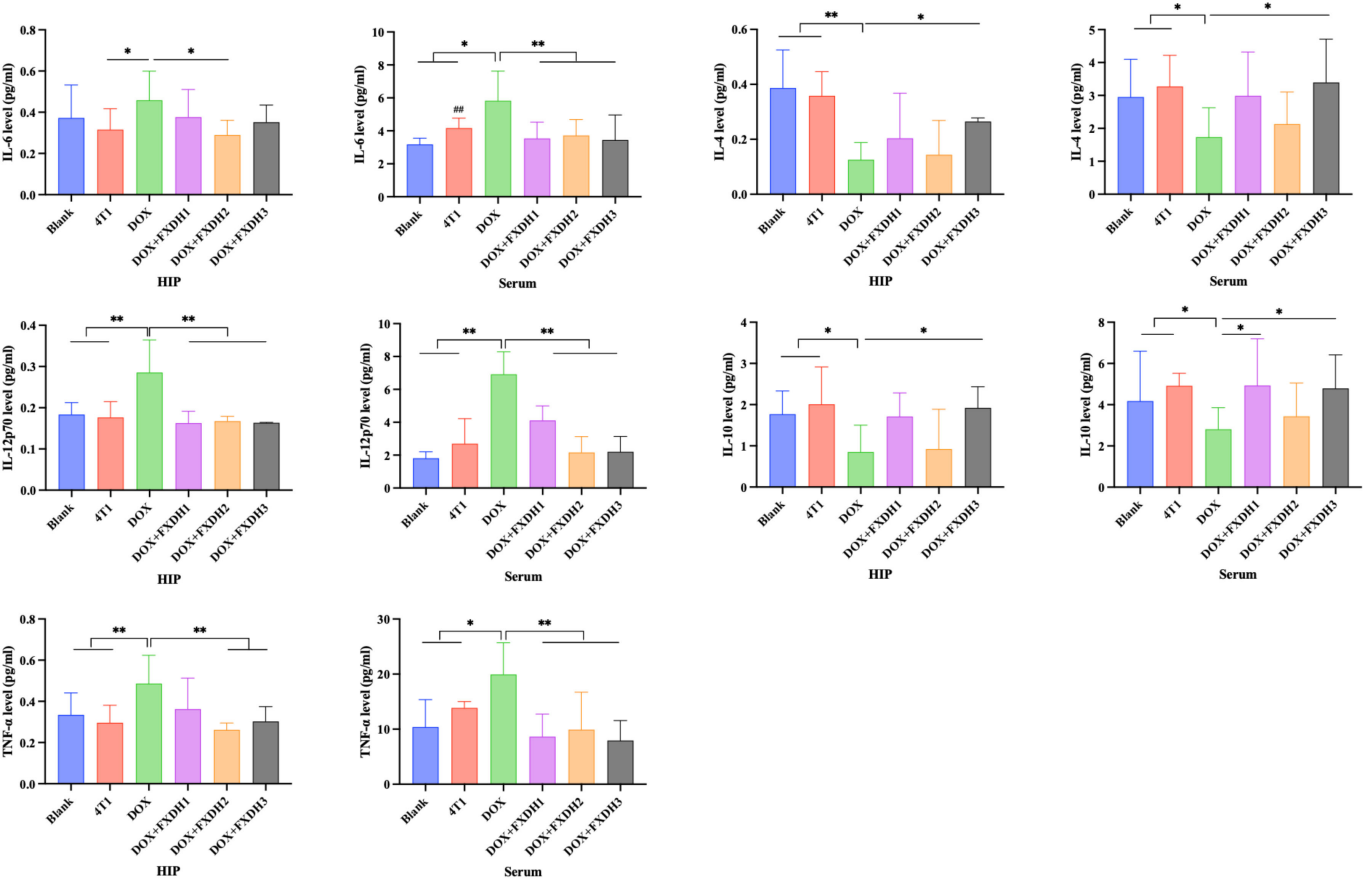
CBA detection of inflammatory cytokine levels in hippocampus and serum (n=8). Hippocampus abbreviated as HIP. * Indicates that compared with the DOX group, * P<0.05, ** P<0.01. ^##^ Indicates that compared with the Blank group, ^##^ P<0.01.

Next, we will use the CBA method to detect cytokine levels in serum and hippocampus, and explore the intervention effect of FXDH on DOX induced inflammation. As shown in **Figure 7**, overall, compared with the Blank group and 4T1 group, DOX treatment increased the levels of pro-inflammatory cytokines IL-6, IL-12p70, and TNF-α in serum and hippocampus (P<0.05). DOX decreased the levels of anti-inflammatory cytokines IL-10 and IL-4 in serum and hippocampus (P<0.05). After FXDH treatment, the increase of pro-inflammatory cytokines and decrease of anti-inflammatory cytokines induced by DOX in serum and hippocampus were improved. Among them, the intervention effect of FXDH medium dose treatment group on pro-inflammatory cytokines was the most significant, while the intervention effect of FXDH low dose on anti-inflammatory cytokines was more significant. Compared with the Blank group, the content of IL-6 in the serum of the 4T1 group significantly increased (P<0.01), while all other indicators changed, but the differences were not statistically significant. This result indicates that in this experiment, tumors can affect the hippocampus and serum inflammatory factors, but the difference is not significant. The experimental results in this section indicate that FXDH could alleviate DOX induced inflammatory responses in serum and hippocampus.

### 3.6 BCA method for detecting protein concentration of oxidative stress markers

**Figure 8:**
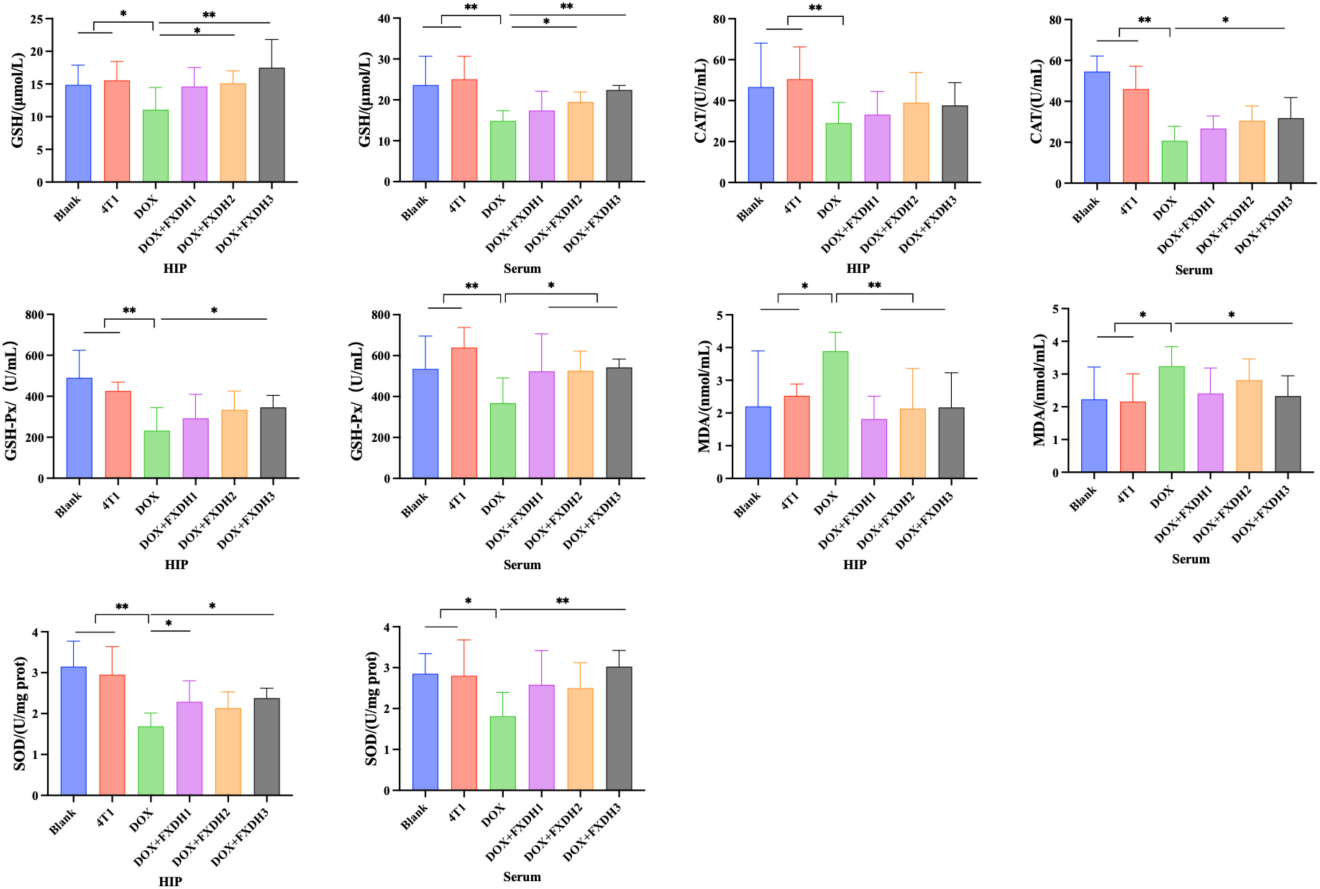
Oxidative stress-related indicators in hippocampus and serum (n=8). Hippocampus abbreviated as HIP. * Indicates that compared to the DOX group, * P<0.05, ** P<0.01.

We applied the BCA method to detect oxidative stress markers in the hippocampus and serum, including GSH, MDA content, as well as GSH-PX, SOD, and CAT. As shown in **Figure 8**, compared with the Blank group, the DOX treatment group showed a significant decrease in GSH content, GSH-PX, SOD, and CAT activity in serum and hippocampus (P<0.05), and a significant increase in MDA levels in serum and hippocampus (P<0.05). Compared with the Blank group, the 4T1 group showed changes in the above indicators, but the differences were not statistically significant.

Compared with the DOX treatment group, the serum and hippocampal GSH levels in the DOX+FXDH2 and DOX+FXDH3 groups significantly increased (P<0.05; P<0.01), while there was no statistical difference observed in the DOX+FXDH1 group. Compared with the DOX treatment group, the GSH-PX activity in the hippocampus of the DOX+FXDH3 group was significantly increased (P<0.05), while the other two groups showed a slight increase, but there was no statistical difference. Compared with the DOX group, the GSH-PX activity in the serum of the DOX+FXDH (1-3) group was significantly increased (P<0.05).

Compared with the DOX treatment group, the MDA levels in the hippocampus of the DOX+FXDH (1-3) group were significantly reduced (P<0.01), and the MDA levels in the serum of the DOX+FXDH3 group were significantly reduced (P<0.05). Compared with the DOX treatment group, the SOD activity in the hippocampus of the DOX+FXDH1 and DOX+FXDH3 groups was significantly increased (P<0.05), and the SOD activity in the serum of the DOX+FXDH3 group was significantly increased (P<0.01), while no statistical differences were observed in the other groups. Compared with the DOX treatment group, there was no statistically significant difference in CAT activity in the hippocampus of the DOX+FXDH (1-3) group, while the CAT activity in the serum of the DOX+FXDH3 group was significantly increased, and the difference was statistically significant (P<0.05).

### 3.7 Nissl staining observation of neuronal morphology

**Figure 9:**
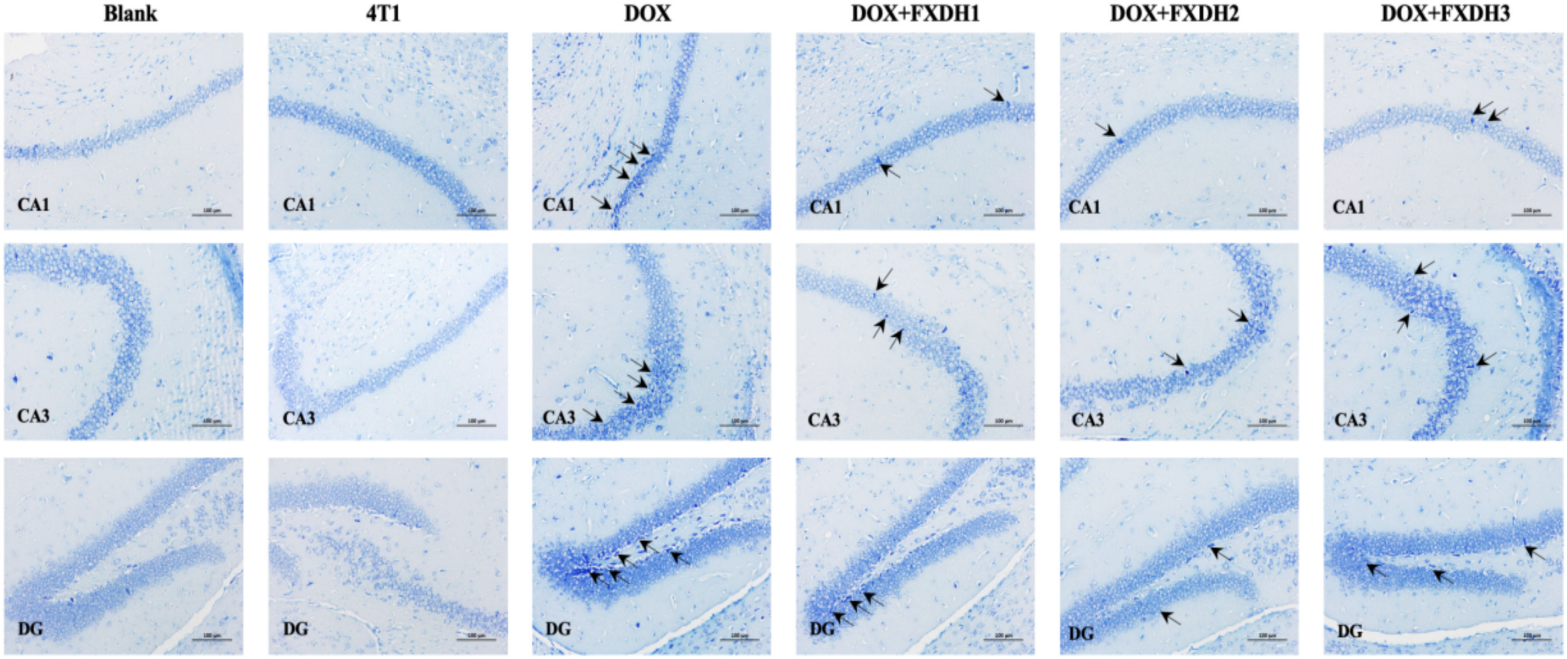
Nissl staining of mouse hippocampus (n=5). Black arrows indicate degenerative changes in neurons in the CA1, CA3, and DG regions. Scale bar: 100 μ m, DG: dentate gyrus.

This study observed the neuronal morphology of mice under different intervention methods through Nissl staining. As shown in **Figure 9**, compared with the Blank group, there were no significant changes in neuronal morphology observed in the 4T1 group. This result indicates that in this experiment, the tumor may not have a significant pathological effect on the hippocampus. Compared with the Blank and 4T1 groups, the DOX group showed more severe degenerative changes in hippocampal CA1, CA3, and DG neurons, manifested as cytoplasmic atrophy and chromatin condensation. The degenerative changes in the DOX+FXDH (1-3) group were reduced compared to the DOX group, with the DOX+FXDH2 group showing the least degree of neuronal morphological degeneration.

### 3.8 Immunofluorescence staining observation of neuroinflammatory changes

To further investigate the intervention effects of DOX and FXDH on neuroinflammation in mice, we used GFAP to observe activated astrocytes in the dentate gyrus (DG) area of the hippocampus, and IBA-1 to observe microglia in the DG area.

**Figure 10:**
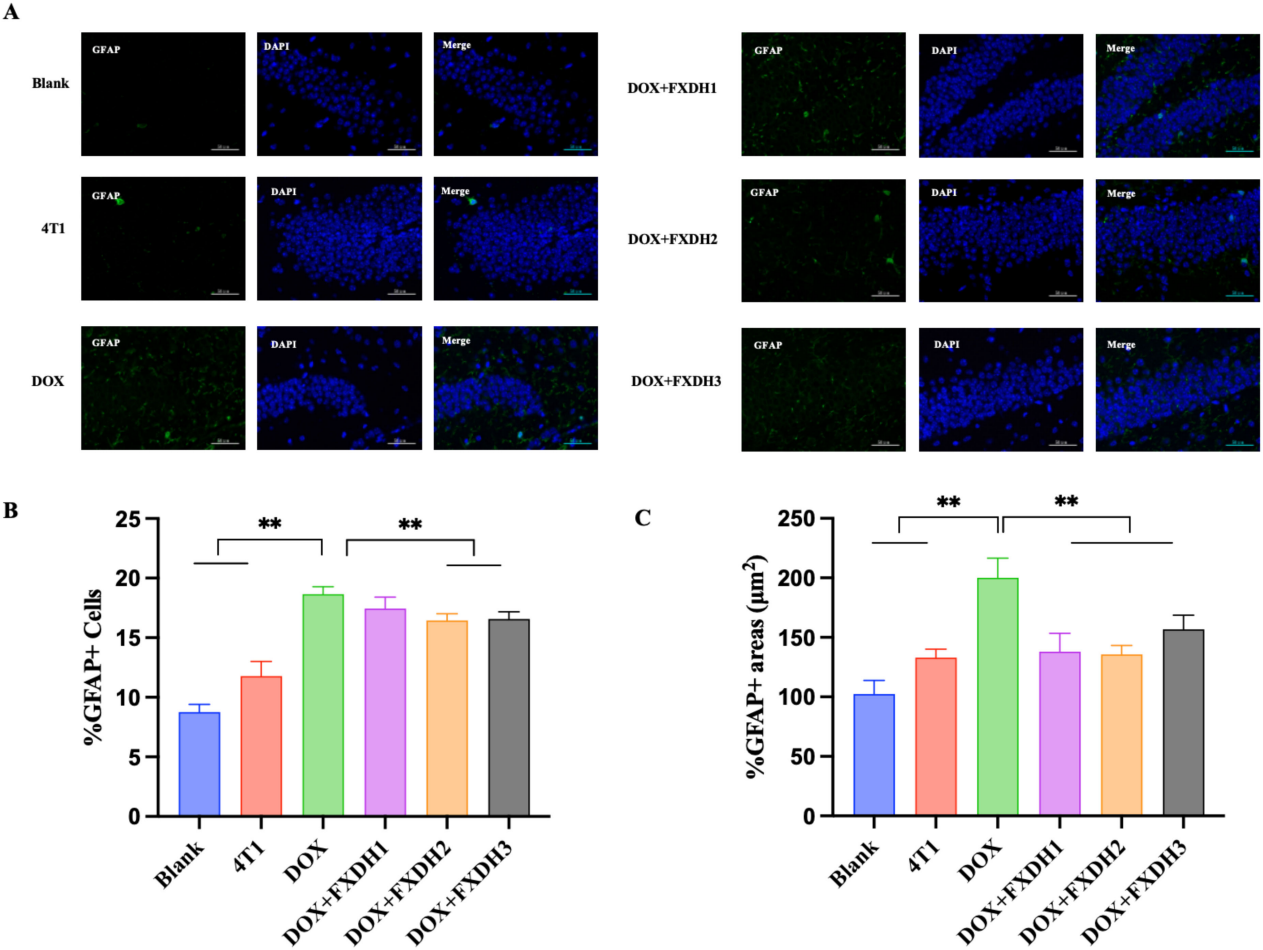
Hippocampus GFAP immunofluorescence staining and data statistics. **(A)** GFAP immunofluorescence staining in hippocampus. GFAP is green, DAPI core counterstain is blue (magnification 400 times), scale bar: 50μm. GFAP: glial fibrillary acidic protein; DAPI: 4’, 6-diamidino-2-phenylindole. **(B)** The number of GFAP labeled astrocytes, ** indicates that compared to the DOX group, ** P<0.01; **(C)** The area of GFAP labeled astrocytes, ** indicates that compared to the DOX group, ** P<0.01.

**Figure 11:**
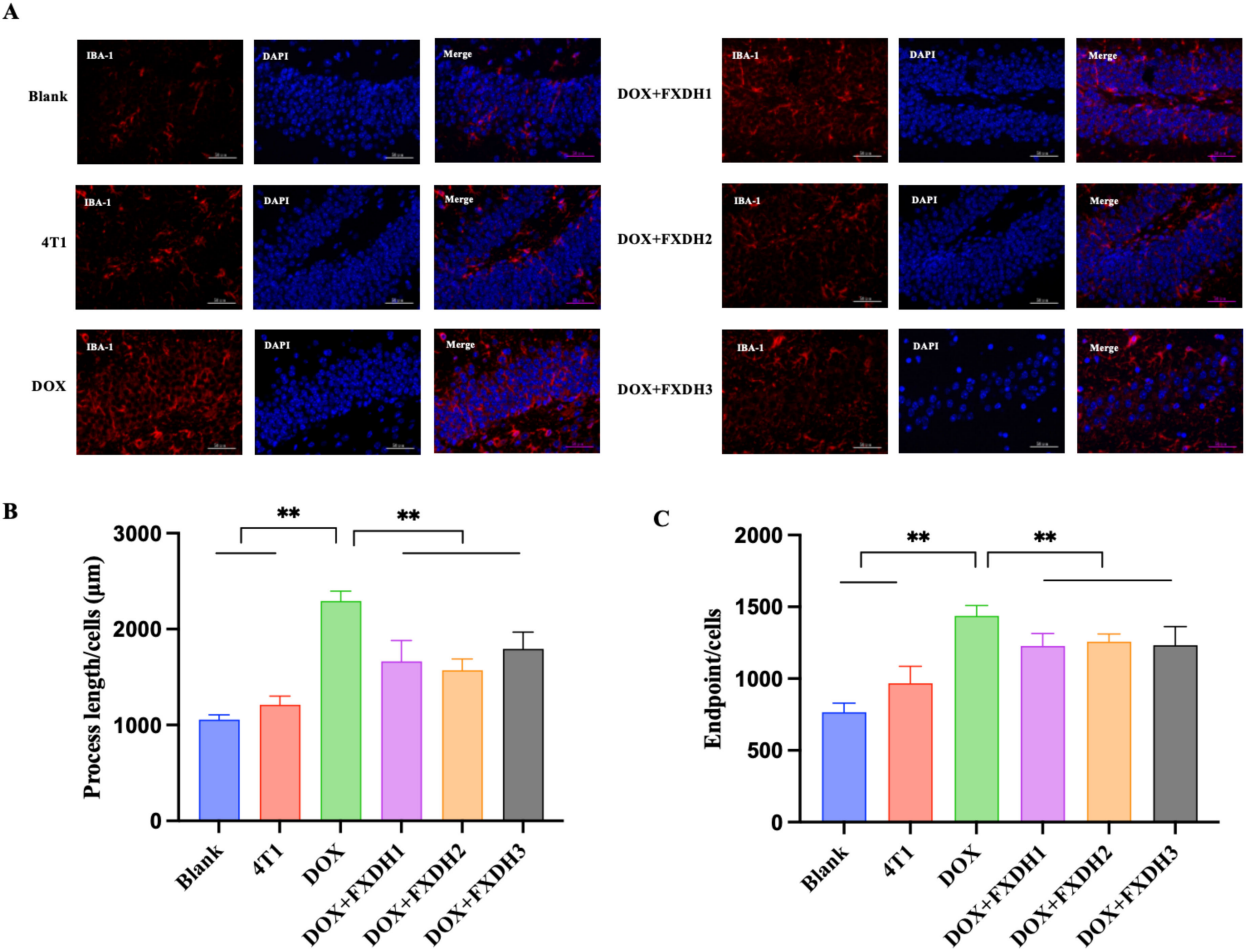
Immunofluorescence staining of IBA-1 in hippocampus and data statistics. **(A)** Immunofluorescence staining of IBA-1 in hippocampus. IBA-1 is red, DAPI nuclear counterstain is blue (magnification 400 times), scale bar: 50µm. IBA-1: Ionized calcium binding adapter molecule; DAPI: 4 ‘, 6- diamidino-2-phenylindole. **(B)** The length of small glial cell processes labeled with IBA-1, ** indicates that compared with the DOX group, ** P<0.01. **(C)** The number of small glial cell endpoints labeled with IBA-1, ** represents the comparison with DOX group, ** P<0.01.

**Figures 10A** and **11A** respectively show Immunofluorescence staining of GFAP and IBA-1 in hippocampus. As shown in **Figures 10**, **11**, compared to the Blank group, the GFAP and IBA-1 immunoreactivity in the dentate gyrus (DG) of the hippocampus in the 4T1 group increased, but the difference was not statistically significant. Compared with the Blank group and 4T1 group, the GFAP and IBA-1 immunoreactivity of the dentate gyrus (DG) in all other groups increased.

As shown in **Figure 10B**, compared with the Blank and 4T1 groups, the number of GFAP labeled astrocytes in the DOX group significantly increased (P<0.01). Compared with the DOX group, the number of astrocytes in the DOX+FXDH2 group and DOX+FXDH3 group significantly decreased (P<0.01). There was no statistical difference in the number of astrocytes between DOX and DOX+FXDH1 groups. As shown in **Figure 10C**, compared with the Blank and 4T1 groups, the area of astrocytes in the DOX group was significantly increased (P<0.01). Compared with the DOX group, the number and area of astrocytes in the DOX+FXDH (1-3) group were significantly reduced (P<0.01), with the DOX+FXDH2 group being the most significant.

As shown in **Figure 11B**, compared with the Blank group and 4T1 group, the protrusion length of IBA-1 labeled microglia in the DOX group significantly increased (P<0.01), while compared with the DOX group, the protrusion length of microglia in the DOX+FXDH (1-3) group significantly decreased (P<0.01). As shown in **Figure 11C**, compared with the Blank group, the DOX group showed a significant increase in the number of small glial cell endpoints (P<0.01). Compared with the DOX group, the DOX+FXDH (1-3) group showed a significant decrease in the length of microglial processes and the number of microglial endpoints (P<0.05).

### 3.9 Blood routine

**Figure 12:**
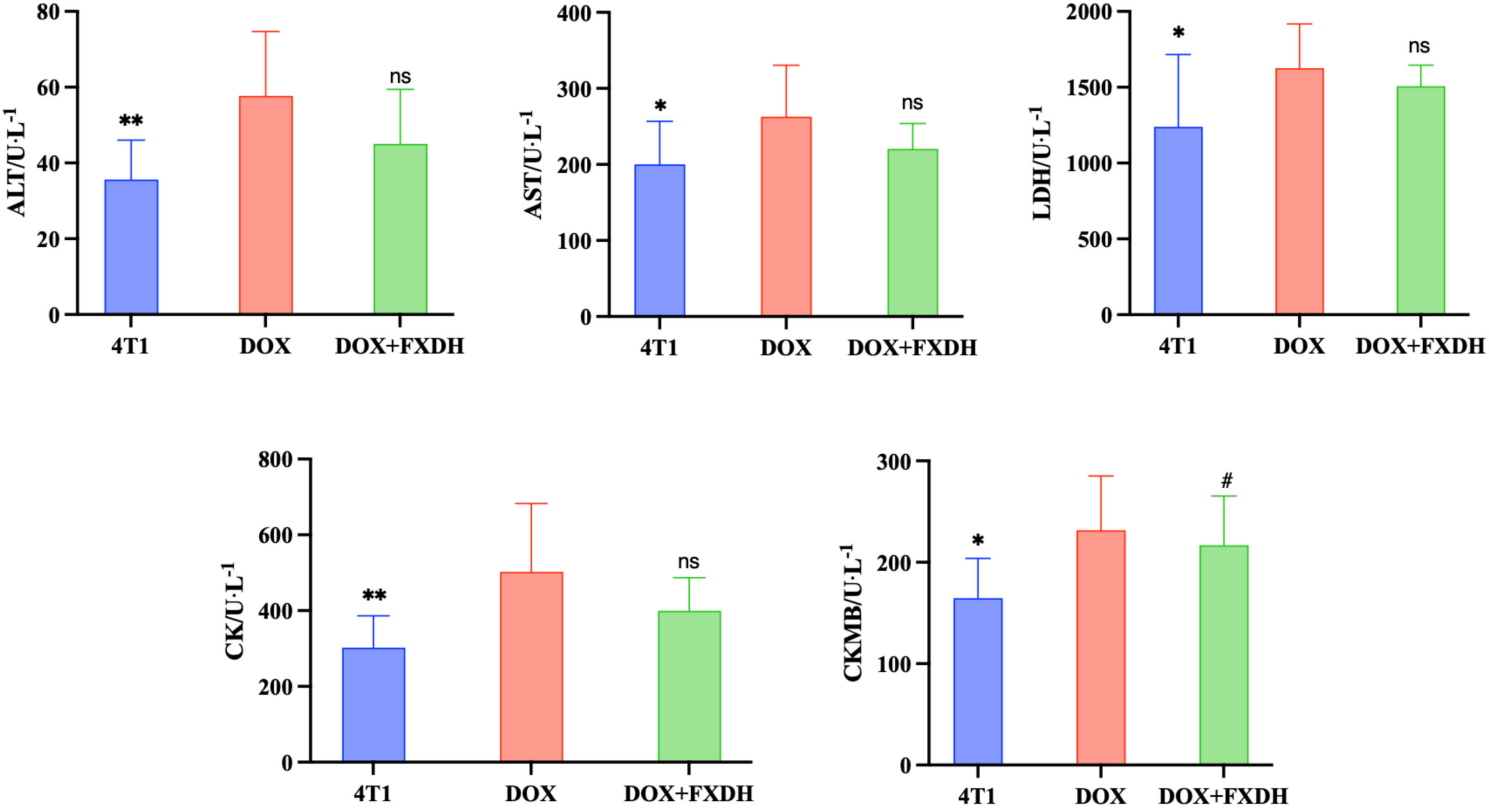
Biochemical examination of blood (n=8). * Indicates that compared with the DOX group, * P<0.05, ** P<0.01, ns represents no statistically significant difference, ^#^Compared with the 4T1 group, ^#^ P<0.05.

After conducting pharmacological studies, we also wanted to explore the toxic side effects of DOX and the intervention effects of FXDH. Based on the above research results, we only selected the medium dose group of FXDH for subsequent experiments. As shown in **Figure 12**, compared with the 4T1 group, the serum levels of ALT, AST, CK, LDH, and CKMB in DOX group mice were significantly increased (P<0.05), and CKMB in DOX+FXDH group mice was significantly increased (P<0.05). Compared with the DOX group, all indicators in the DOX+FXDH group of mice were reduced, but there was no statistical difference.

### 3.10 Evaluation of myocardial tissue pathological damage by cardiac HE staining

**Figure 13:**
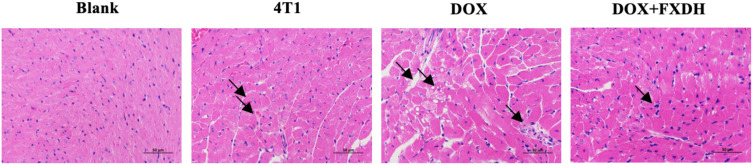
Pathological changes in myocardial tissue. Only the FXDH medium dose group was selected for HE staining. The arrow indicates mild swelling and uneven staining of myocardial cells, suggesting inflammatory infiltration HE staining, 400x, scale bar: 50μm.

As shown in **Figure 13**, the HE staining boundary of the blank group heart is clear, and the staining of myocardial cells is uniform. The 4T1 group showed mild swelling of myocardial cells and mild local inflammatory response. The DOX group had disordered arrangement of myocardial cells, uneven cytoplasmic staining, and inflammatory infiltration of myocardial tissue. Compared with the DOX group, the DOX+FXDH group had less inflammatory infiltration and mild swelling of myocardial cells.

## 4 Discussion

In this study, consistent with previous research results, DOX can promote the expression of pro-inflammatory cytokines IL-6, IL-12p70, and TNF-α in the hippocampus and serum of mice, and reduce the expression of anti-inflammatory cytokines IL-4 and IL-10 in the hippocampus and serum. After the treatment of FXDH, the inflammatory response induced by DOX was alleviated, with the medium and low dose groups of FXDH showing better efficacy. Oxidative stress is the main link in chemotherapy induced CRCI and is associated with the progression of many neurodegenerative diseases, such as Parkinson’s disease, Alzheimer’s disease, etc. (23, 70). Due to the high demand for oxygen in the brain, it is highly susceptible to oxidative stress (24, 71). Existing research has found that anthracycline drugs (such as DOX) can produce high levels of ROS (4, 72, 73), and the generation of oxidative stress in the brain may lead to mitochondrial dysfunction, activation of glial cells, triggering of programmed cell death, and proteasome dysfunction (74, 75). Studies have shown that intraperitoneal injection of DOX for 4 weeks in rats resulted in an 80% decrease in manganese superoxide dismutase (MnSOD) levels in hippocampal homogenate (76), while combined administration of DOX and cyclophosphamide for 3 weeks in rats showed an increase in 8-oxodg immunoreactivity and GPx1 levels in the hippocampus (77).

Peripheral anticancer drugs (such as DOX) trigger oxidative stress, leading to protein oxidation and lipid peroxidation, such as apolipoprotein 1 (Apo-1) oxidation. This oxidation leads to a large production of pro-inflammatory cytokines. Inflammatory cytokines can cross the BBB through passive diffusion or receptor-mediated endocytosis. Once in the brain, pro-inflammatory cytokines trigger an immune response, promoting the production of more pro-inflammatory cytokines by astrocytes, microglia, and NF-κB in the hippocampus. Therefore, the expression of inducible nitric oxide synthase (iNOS) increases, triggering an increase in ROS, reactive nitrogen species (RONS), and oxidative stress in the brain, which in turn leads to DNA damage and damage to several proteins (such as MnSOD), making it unable to protect neuronal cells from oxidative stress and exacerbating the cycle (4). In addition, even without entering the brain, pro-inflammatory cytokines can damage the BBB by disrupting the tight junctions of BBB microvascular endothelial cells and increasing their permeability (78). The results of Nissl staining and immunofluorescence showed that FXDH can improve DOX induced neurodegeneration and neuroinflammation, with the FXDH medium dose group showing a more significant relative effect. In this study, DOX increased the proliferation and overexpression of GFAP^+^ and IB-1^+^ cells in hippocampal tissue, indicating that DOX promotes the activation of astrocytes and polarization of microglia. After FXDH intervention, this phenomenon was alleviated. The above results indicate that DOX induces dysregulation of inflammatory responses in the periphery and brain.

At the same time, under the experimental conditions, tumors also had negative effects on the cognitive function, neuroinflammation, and oxidative stress of mice. However, compared with the blank group, the differences in various indicators were not significant. Therefore, in this study, the neuroinflammation of mice was mainly caused by DOX. Several factors may contribute to the development of CRCI, such as age, genetic susceptibility, psychological and sociodemographic factors (40). Studies have shown that compared with women under the age of 65, breast cancer patients over the age of 65 are more likely to have a decline in cognitive ability (79), and the anxiety (80–83), post-traumatic stress disorder (84, 85), sleep difficulties (fatigue and insomnia) (9) of tumor patients are related to cognitive impairment. Current studies have found that the cognitive impairment of cancer patients may be caused by cancer treatment or the existence of cancer itself (40). In a prospective, longitudinal, controlled study, 289 patients with colorectal cancer and 136 healthy controls were included to evaluate the impact of tumor itself and chemotherapy on cognition. The results showed that the diagnosis of tumor would lead to severe cognitive impairment, which lasted for 2 years, while the effect of chemotherapy on cognitive function was not significant (9). In this study, we set up a 4T1 group to evaluate the impact of tumor itself on cognitive function. However, due to the limitations of animal experiments, we could not accurately determine whether the tumor itself will damage cognition. This also requires further clinical trials to evaluate the age, psychological status, quality of life, treatment options and other dimensions to make a clear diagnosis.

Y maze and NOR behavioral experiments were used to observe changes in cognitive behavior in mice. Through literature search, we found that the Morris water maze (MWM) experiment is most commonly used for evaluating animal cognitive function (86, 87). However, in the preliminary experiment, we found that 21 days after the successful construction of the mouse breast cancer transplantation tumor model, the tumor would have a greater load on the body of the mice, which made it difficult for them to complete the MWM, so we replaced the Y maze experiment for detection.

Research has shown that oxidative stress, neuroinflammation, and neurodegenerative diseases (such as Alzheimer’s disease, cognitive impairment, etc.) in the brain are closely related (88). In the process of oxidative stress, the decrease of GSH and the generation of excessive ROS will produce MDA, while SOD, CAT, etc. have a positive effect on reversing oxidative stress. In this study, the results showed that FXDH intervention reversed to varying degrees the changes in oxidative stress markers in the hippocampus and serum induced by DOX. In this part of the experiment, overall, the low-dose FXDH showed the most significant improvement in DOX induced oxidative stress. The cause of this phenomenon is currently unclear, and even after repeated experiments, similar conclusions can still be drawn. The high-dose FXDH group did not show optimal results, and our analysis suggests that this may be due to the complex composition of TCM decoction and the non-linear relationship between dosage and efficacy. At low to moderate doses, the active ingredient may reach its optimal concentration and exert therapeutic effects; High doses may lead to excessive ingredients, causing side effects and weakening the therapeutic effect. In addition, we speculate that high doses may increase the metabolic burden on animals, affect the absorption, distribution, and excretion of drugs, reduce the bioavailability of active ingredients, and thus weaken the therapeutic effect. We will further explore its reasons.

DOX, as a common anthracycline antibiotic, is often limited in clinical practice due to its adverse reactions (89, 90). Among them, DOX induced cardiotoxicity and hepatotoxicity are the most common adverse reaction (89, 91, 92). We used blood routine and cardiac HE staining to observe the side effect of DOX, as well as the intervention effect of FXDH. The blood routine results showed that, FXDH did not significantly alleviate the cardiac toxicity induced by DOX. The cardiac HE results showed that FXDH could alleviate the inflammatory infiltration induced by DOX.

## 5 Limitations of the study

1. This study only carried out experiments based on murine model to explore the efficacy of FXDH in the intervention of DOX induced CRCI, which could not explain the clinical efficacy and application prospect of FXDH. Pharmacological and toxicological experiments should be carried out to determine its safety, and clinical experiments should be carried out to verify the efficacy in patients with breast cancer.
2. Due to the limited survival time of breast cancer bearing mice, This study attempted to simulate clinical application scenarios as much as possible, and we cannot observe the effect of FXDH on long-term cognitive impairment. In addition, only a few studies have explored the impact of tumor itself (without chemotherapy) on cognition. Although 4T1 group is set up in this research, it is also not convincing. The correlation between tumor and CRCI may be related to the psychological state of patients in the process of knowing the disease and treatment.
3. After the end of this experiment, we selected the classic cognitive related pathway SIRT1/NF-κB to explore the specific mechanism of FXDH (62). We studied the effect of FXDH on the expression of SIRT1/NF-κB key proteins in the hippocampus and the effect on the genes related to microglia polarization and used rats to prepare FXDH containing serum and HT-22 neuron cells to study the mechanism at the cellular level, but no positive conclusion was obtained. As the result, this part of the content is not shown in the research. We will further study the mechanism in the follow-up work.
4. This study only observed that FXDH can alleviate CRCI caused by DOX, but the degree of damage to the BBB caused by DOX and how FXDH achieves protective effects are not yet clear. In the future, rats can be used for experiments to detect relevant indicators of the BBB and cerebrospinal fluid.

## 5 Conclusion

In summary, in the mouse model, FXDH has the effect of anti-cognitive impairment after chemotherapy for breast cancer, and can improve the DOX induced learning, memory and cognitive impairment in mice. FXDH can reverse DOX induced neuroinflammation by improving the neurodegenerative changes caused by DOX, reducing pro-inflammatory cytokine levels in mouse serum and hippocampus, increasing anti-inflammatory cytokine levels, and reducing oxidative stress response.

## Data Availability

The original contributions presented in the study are included in the article/**Supplementary Material**, further inquiries can be directed to the corresponding author/s.
