# Supplementary material for: Effect of Fangxia-Dihuang Decoction on doxorubicin-induced cognitive impairment in breast cancer animal model
Source: Front Oncol. 2025 Apr 28;15:1515498. doi: 10.3389/fonc.2025.1515498 (PMC12066564; doi:10.3389/fonc.2025.1515498)
Supplement: Supplementary file 1 [file DataSheet1.docx]

Supplementary materials 2. Weight changes of mice in each group (x ± s, unit: g, n=6)

| Grouping | Day 0 | Day 7 | Day 14 | Day 21 | Day 28 |
| --- | --- | --- | --- | --- | --- |
| Blank | 18.09±0.77 | 18.44±0.72 | 18.83±0.50 | 19.05±0.45 | 19.44±0.64 |
| 4T1 | 18.54±0.80 | 18.83±0.71 | 18.29±0.70 | 18.15±0.47^*^ | 18.10±0.44^*^ |
| DOX | 18.59±0.62 | 19.00±0.81 | 17.98±0.60^*^ | 17.45±0.51^*^ | 17.60±0.58^*^ |
| DOX+FXDH1 | 18.42±1.20 | 18.68±1.12 | 17.81±0.97^*^ | 17.87±1.00^*^ | 17.30±0.70^*△^ |
| DOX+FXDH2 | 18.14±0.60 | 18.71±0.48 | 18.18±0.41 | 17.99±0.35^*^ | 17.88±0.24^*^ |
| DOX+FXDH3 | 18.05±0.57 | 18.55±0.53 | 17.90±0.60^*^ | 17.51±0.58^*^ | 17.33±0.44^*△^ |

*Compared with the Blank group, * *P*<0.05; △ indicates that compared with the 4T1 group, △ *P*<0.05.

Supplementary materials 3. Changes in tumor volume of tumor bearing mice (x ± s, unit: mm^3^, n=5)

| Days | 4T1 | DOX | DOX+FXDH1 | DOX+FXDH2 | DOX+FXDH3 |
| --- | --- | --- | --- | --- | --- |
| Day 6 | 16.00 ± 2.02 | 16.13 ± 2.71 | 15.99 ± 3.07 | 16.44 ± 2.32 | 15.77 ± 3.17 |
| Day 9 | 60.54 ± 8.91 | 56.78 ± 14.11 | 56.33 ± 11.28 | 59.59 ± 10.25 | 62.66 ± 13.30 |
| Day 12 | 161.41 ± 40.71 | 126.57 ± 33.52 | 106.03 ± 22.65^*^ | 106.71 ± 36.94^*^ | 130.07 ± 36.37 |
| Day 15 | 203.53 ± 57.00 | 158.25 ± 33.71 | 127.48 ± 26.93^*^ | 148.08 ± 48.66^*^ | 164.75 ± 34.93 |
| Day 18 | 286.45 ± 68.59 | 246.75 ± 72.86 | 256.31 ± 81.20 | 228.04 ± 74.03 | 245.70 ± 81.18 |
| Day 21 | 422.32 ± 89.60 | 340.37 ± 90.12 | 347.00 ± 79.23 | 307.51 ± 87.36^*^ | 314.13 ± 102.73^*^ |
| Day 24 | 622.44 ± 93.26 | 422.30 ± 84.00^*^ | 391.94 ± 98.47^*^ | 431.93 ± 73.21^*^ | 392.96 ± 81.57^*^ |
| Day 27 | 871.75± 112.78 | 698.18 ± 134.26^*^ | 658.51 ± 131.01^*^ | 580.66 ± 78.15^*^ | 671.13 ± 119.74^*^ |

Note: * indicates that compared to the 4T1 group, * P<0.05.

Supplementary materials 4. Tumor weight of tumor bearing mice (n=5)

Note: On the 35th day of the experiment, we euthanized the mice and then weighed the tumor tissue. * indicates that compared with the 4T1 group, * *P*<0.05, ns represents no statistically significant difference.


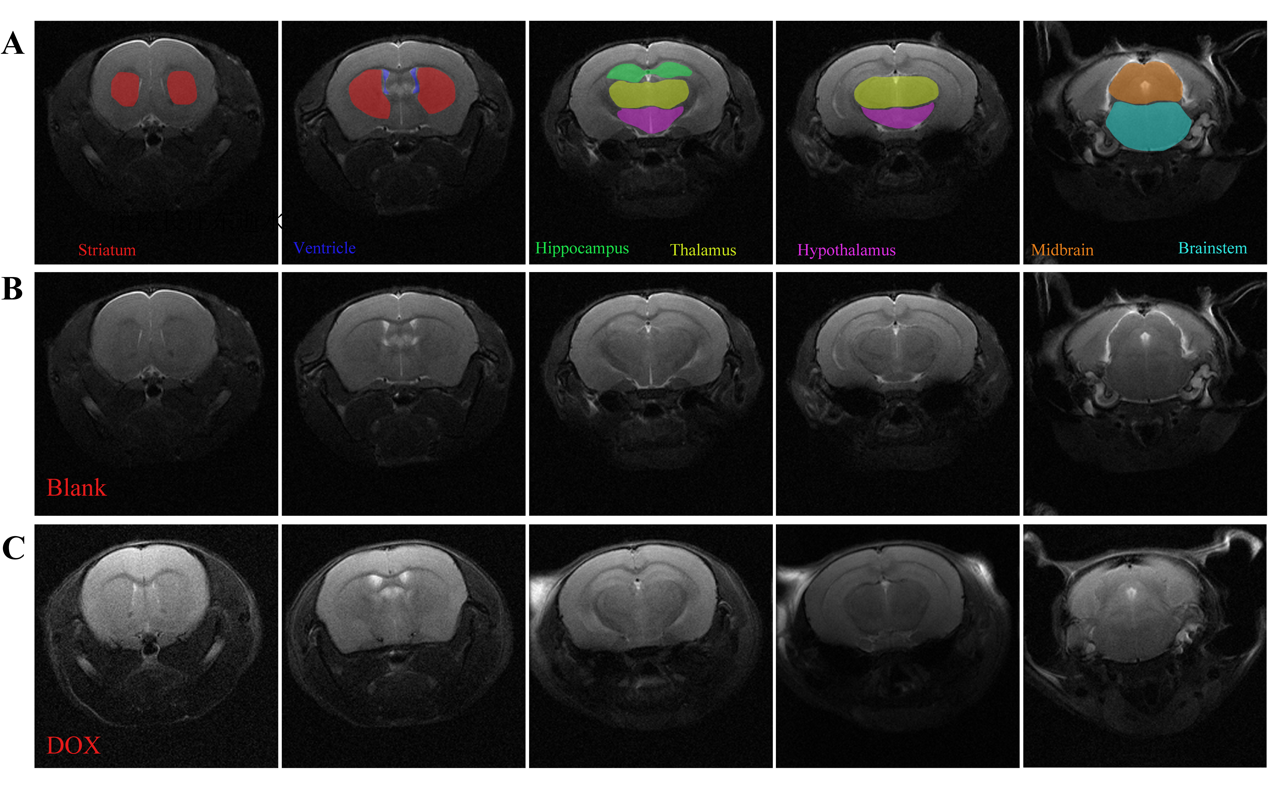


Supplementary materials 5. T2 weighted image of mouse brain MRI scan

Note: (A) Schematic diagram of the position of striatum, ventricle, hippocampus, thalamus, hypothalamus, midbrain and brainstem; (B) Representative schematic diagram of T2 weighted images at different brain levels of blank group mice; (C) Representative schematic diagram of T2 weighted images at different brain levels of mice in DOX group.

Supplementary materials 6. The dose selection involved in this study

The combination therapy group was given a solution of FXDH freeze-dried powder by gavage. The high-dose group was treated with 19 g/kg ·d of crude drug, the medium dose group was equivalent to 13.65 g/kg·d of crude drug (clinical equivalent dose), and the low-dose group was equivalent to 6.83 g/kg·d. The main reasons for choosing this dose in the high-dose group are as follows:

1. We found in the preliminary experiment that even with ultrasound assisted dissolution and other methods, twice the medium dose (equivalent to 27.3 g/kg·d of raw medicine) of FXDH freeze-dried powder still could not be fully dissolved. After gradually reducing the concentration of herbs, we chose 19 g/kg·d for intervention.
2. In the preliminary experiment, in order to maintain the original therapeutic concentration of the high-dose group, we intervened by gavage twice in the morning and evening. However, the high-dose group mice showed significant weight loss and reduced food intake after administration, so we ultimately abandoned this intervention method. The reason for this phenomenon may be related to the fragility of the esophagus in mice, and increasing the frequency of gastric lavage may cause certain damage to the esophagus, thereby affecting the normal feeding of mice.

In the preliminary experiment, we attempted various dosages and frequencies of DOX administration. Firstly, we did not observe any significant cognitive impairment in mice during behavioral experiments after three consecutive administrations starting from 2mg/kg. We gradually increased the dosage to 5mg/kg, and after 3 doses, the mice showed significant abnormalities in the Y maze experiment. Subsequently, we randomly selected a mouse for MRI brain testing and found that DOX mice exhibited brain edema (see Supplementary materials 5). In terms of dosing frequency, due to the limited survival cycle of tumor bearing mice, we were unable to administer the fourth dose. In addition, we attempted to administer 2mg/kg at intervals of 1 and 2 days, but in the second week, the mice showed obvious irritability and were even unable to be grasped. Finally, we chose a weekly intraperitoneal injection of 5mg/kg. The scientific validity of the dosage of the medication needs further verification.
